# Overweight and obesity in children with congenital heart disease: combination of risks for the future?

**DOI:** 10.1186/1471-2431-14-271

**Published:** 2014-10-16

**Authors:** Sandra Mari Barbiero, Caroline D’Azevedo Sica, Daniela Schneid Schuh, Claudia Ciceri Cesa, Rosemary de Oliveira Petkowicz, Lucia Campos Pellanda

**Affiliations:** Post-Graduation Program in Health Sciences: Cardiology, Instituto de Cardiologia/Fundação Universitária de Cardiologia, Porto Alegre, Brazil; Post-Graduation Program in Human Movement Sciences from the Universidade Federal do Rio Grande do Sul, Porto Alegre, Brazil; Universidade Federal de Ciências da Saúde de Porto Alegre, Avenida Princesa Isabel, 370, Santana, Postal Code: 90620-000 Porto Alegre, RS Brazil

**Keywords:** Child, Adolescent, Congenital heart disease, Overweight, Ischemic disease

## Abstract

**Background:**

Children who have unhealthy lifestyles are predisposed to develop hypertension, dyslipidemia and other complications. The epidemic of obesity is also affecting children with congenital heart disease. The aim of this study is to estimate the prevalence of obesity and describe associated risk factors, including family history in children with congenital heart disease.

**Methods:**

A cross-sectional study with 316 children and adolescents with congenital heart disease seen in an outpatient clinic of a reference hospital. Collected sociodemographic data included family history of chronic disease, dietary habits, laboratory tests (total cholesterol, HDL and LDL/cholesterol, triglycerides, fasting glucose, CRP, hematocrit and hemoglobin), and anthropometric assessment. Anthropometric data of the caregivers was self-reported.

**Results:**

The prevalence of excess weight was 26.9%. Altered levels of total cholesterol were observed in 46.9%, of HDL in 32.7%, LDL in 23.6% and of triglycerides levels in 20.0%. A higher frequency of family history of obesity (42.6%; p = 0.001), dyslipidemia (48.1%; p = <0.001), diabetes (47.4%; p = 0.002), hypertension (39.2%; p = 0.006) and ischemic disease (43.7%; p = 0.023), as well as significantly higher values of triglycerides (p = 0.017), glycemia (p = 0.004) and C-reactive protein (p = 0.002) were observed among patients with excess weight.

**Conclusion:**

The presence of modifiable risk factors and the variables associated to excess weight in this population was similar to that described in the literature for children without congenital disease. As these children already present the risks associated to heart disease, it is particularly important to promote a healthy lifestyle in this group.

## Background

During the last three decades, there has been a considerable increase in the prevalence of obesity in children and adolescents (4–18 year-old) worldwide [1–3]. Children and adolescents with unhealthy lifestyles are predisposed to develop hypertension, dyslipidemia and other complications [4]. These factors, as well as physical inactivity, may track into adulthood [5] and increase the risk of chronic diseases such as atherosclerosis [1].

The epidemic of obesity is also affecting children with congenital heart disease (CHD). More than one quarter of this population is already overweight [6, 7]. Two main causes have been described: physical activity restrictions and interventions for weight gain in infancy, when many lesions cause undernutrition [5]. These interventions often include consumption of increased calories and foods with high fat and sodium content [8, 9]. Although nutritional requirements and physical functional capacity change as these children grow older and their heart lesions are successfully treated, the inappropriate dietary behaviors and physical inactivity are frequently maintained across childhood [10]. The family frequently influences these unhealthy behaviors, both directly, restricting physical activity, for example, and indirectly, by setting an unhealthy model. When parents are obese, as one example, the risk of obesity in their children is increased [11–14].

Therefore, the objective of the present study was to estimate the prevalence of overweight, obesity and associated physical activity habits, passive smoking, glycemia and lipids in children with congenital heart disease. We also sought to investigate cardiovascular risk factors present in children’s families.

## Methods

We conducted a cross-sectional study of 316 patients with congenital heart disease, aged between 2 and 18 years, and receiving outpatient care at the Pediatric Cardiology Outpatient Clinic of a referral hospital between September 2010 and March 2013. The study protocol was approved by the Institutional Research Ethics Committee of Instituto de Cardiologia do Rio Grande do Sul, Brazil (4470/2010).

Patients who had innocent murmur, clinical conditions that prevented anthropometric assessment (wheelchair users, malformation of the lower limbs, etc.), genetic syndromes or children without a diagnosis of structural heart disease were excluded from the study.

Data collection was performed according to a weekly list of patients scheduled for routine outpatient visits. Based on this list, the children’s guardians were contacted by telephone, and the patients were invited to participate in the study. Those who accepted to participate were asked to fast for 12 hours before laboratory tests. Patients who could not be reached by phone were invited to participate during the medical visit, and their laboratory tests were scheduled for another day.

All patients and guardians received information about the study and, after accepting to participate, signed the written consent form. Next, patients underwent collection of blood samples and anthropometric assessment. The participants’ caregivers present during data collection provided information about family risk factors and physical activity habits (International Physical Activity Questionnaire-IPAQ short version) [15]. Data were collected using a questionnaire administered by health professionals who attended two specific training sessions and received training updates regularly. After being assessed, participants who showed abnormal results were referred to multidisciplinary outpatient care for prevention and treatment of risk factors.

Weight was measured to the nearest 0.1 kg and height to the nearest centimeter using a Welmy electronic digital scale with stadiometer, with 200 Kg capacity, with the child standing, without shoes or heavy clothing. Nutritional status was based on body mass index (BMI), and classified using the software WHO Anthro and Anthro Plus. Cutoff points for underweight/normal weight (<85th percentile) and excess weight (>85th percentile being overweight 85th −95th percentile and obesity > 95 percentile) for BMI values were used according to the WHO-2006/2007 [16].

Blood was collected by peripheral venous puncture after 12 h fasting. The hematocrit and hemoglobin were determined using whole blood collected with ethylenediaminetetraacetic acid (EDTA), in an automated analyzer (Coulter Act, Coulter, USA). Biochemical analysis of total cholesterol, LDL, HDL cholesterol and triglycerides were determined in serum obtained by centrifugation of blood samples, through enzymatic method on an automated analyzer (Selectra E, Vital Scientific, USA), using reagent kits and protocols according to instructions of the manufacturer. Levels of hs-CRP were determined in serum by nephelometry, using a Behring Nephelomefer 100 Analyzer (Dade Behring, USA).

Blood tests were considered abnormal according to the U.S. pediatric guidelines (2011) and the I Brazilian Guidelines for Prevention of Atherosclerosis in Childhood and Adolescence (2005): total cholesterol > 170 mg/dL, HDL/cholesterol < 45 mg/dL, LDL/cholesterol > 110 mg/dL, triglycerides > 75 mg/dL (2–9 years) or > 90 mg/dL (10–18 years) [17], fasting glucose > 100 mg/dL, CRP > 0.30 mg/dL, hematocrit < 35%, and hemoglobin < 11.0 g/dL [18].

Sample size was estimated as 250 children and adolescents, based on the prevalence of obesity observed in a previous study [19], with absolute error margins ranging from 3% to 6% with a confidence level of 95%.

Data were stored and analyzed using the computer program SPSS, version 17.0. The prevalence rates were expressed as percentages with 95% confidence intervals. The association between risk factors was assessed using the chi-square test or Fisher’s exact test. Differences between the groups with and without risk factors were evaluated using the Student *t* test or Mann–Whitney test for continuous variables and the chi-square test or Fisher’s exact test for categorical variables (gender, total cholesterol, HDL/cholesterol, LDL/cholesterol, triglycerides, hematocrit, hemoglobin, glucose, BMI percentile). Poisson multiple logistic regression analysis was adjusted for family history (obesity, dyslipidemia, diabetes, hypertension, and ischemic heart disease), mother’s nutritional status, both parents’ nutritional status, and adolescents’ age. Statistical significance was set at p-value ≤ 0.05.

This report is presented as suggested by the STROBE statement: guidelines for reporting observational studies [20].

## Results

A total of 341 patients were interviewed, but 25 did not collect blood and were excluded from analysis, resulting in 316 participants . Most participants were male (55.7%), Caucasian (81.6%) and aged between 6 and 11 years (43.7%). The majority had been born at term (83.2%) and had acyanotic congenital heart disease (81,1%). The proportion of passive smoking was reported to be 43.7% (Table 1).

**Table 1:** **General characteristics of the population**

Variables	n = 316 (%)
Male	176 (55.7)
White	258 (81.6)
Age	
Preschool age (2–5 years)	67 (21.2)
School age (6–11 years)	138 (43.7)
Adolescents (12–18 years)	111 (35.1)
Born at term	263 (83.20)
Heart disease	
Acyanotic	
Ventricular Septal Defect (VSD)	76 (24.1)
Atrial Septal Defect (ASD)	61 (19.3)
Miscelaneous	119 (37,7)
Cyanotic	
Tetralogy of Fallot	43 (13.6)
Pulmonary Atresia	6 (1.9)
Miscelaneous	11 (3,4)
Father’s educational level	
Elementary school	191 (68.0)
High school	79 (28.1)
Incomplete/Complete higher education	11 (3.9)
Mother’s educational level	
Elementary school	184 (60.7)
High school	94 (31.0)
Incomplete/Complete higher education	25 (8.3)
Number of siblings	
Only child	67 (21.2)
Siblings	249 (78.8)
Positive family history for	
Excess weight	140 (44.3)
Dyslipidemia	170 (53.8)
Diabetes	157 (49.7)
Hypertension	263 (83.2)
Heart disease/ischemia	165 (52.2)
Presence of smokers in the household	138 (43.70)

Family history of cardiovascular risk factors included excess weight in 44.3%, dyslipidemia in 53.8%, diabetes in 49.7%, arterial hypertension in 83.2%, and ischemic disease in 52.2% (Table 1).

The prevalence of excess weight (BMI ≥ 85th percentile) was 26.9%; of these, 17.4% were overweight (BMI > P85 ≤ 95) and 9.5% were obese (BMI > P95). Excess weight was more common among boys (60%). In the group of 6–11 years old, 34.1% presented with excess weight (p = 0.009). The group of acyanotic congenital heart disease showed 27.7% of overweight, while in patients with cyanotic lesions the proportion was 23,3 (Table 2).

**Table 2:** **Distribution of general characteristics of the population according to the BMI classification of individuals with congenital heart disease**

Variables	Total 316 (%)	Underweight/Normal weight 231 (%)	Excess weight 85 (%)	PR	CI (95%)	p
Male	176 (55.7)	125 (54.1)	51 (60)	1,20	0.84-1.74	0.321
Only child	67 (21.2)	49 (21.2)	18 (21.2)	1.0	0.65-1.55	0,99
Preterm birth	49 (15.5)	31 (13.4)	18 (21.2)	1.53	1.01-2.32	0.046
Age						
Preschool age (2–5 years)	67 (21.2)	57 (24.7)	10 (11.8)	1.0	-	-
School age (6–11 years)	138 (43.7)	91 (39.4)	47 (55.3)	2.29	1.24-4.23	0.008
Adolescent (12–18 years)	111 (35.1)	83 (35.9)	28 (32.9)	1.71	0.89-3.27	0.104
Congenital heart disease						
Cyanotic	60 (19)	46 (19,9)	14 (16,5)	0,84	0,51-1,37	0,48
Acyanotic	256 (81)	185 (80,1)	71 (83,5)	1,0	-	-

PR: prevalence ratio; CI: confidence interval.

Regarding physical activity classification, children and adolescents with excess weight were very active in 20%, active in 36.5% and irregularly active in 40%, while eutrophic children were very active in 19.1%, active in 38.7% and irregularly active in 35.7% (p = 0.802).

There were 165 mothers (52,2%), and 92 fathers (29,1%) with excess weight. Mothers’ and both parents’ excess weight was significantly associated with children’s excess weight (p = 0.003 and 0.049, respectively). The Prevalence Ratio of an excess weight mother to have an excess weight child was 1.24 (CI 1.08-1.43).

As shown in Table 3, the excess weight group had more often a positive family history (first degree relative) for obesity (p = 0.002), dyslipidemia (p = <0.001), diabetes (p = 0.005), hypertension (p = 0.010), and ischemic disease (p = 0.040). The prevalence ratio for excess weight in children was 1.92 (CI 1.22 ± 3.02, p = 0.005) when the mother had excess weight and 1.74 (CI 1.15 ± 2.62; p = 0.009) when there was a positive family history for dyslipidemia.

**Table 3:** **Family history of obesity and chronic diseases according to the BMI categories of individuals with congenital heart disease**

Variables	Total 316 (%)	Underweight/Normal weight 231 (%)	Excess weight 85 (%)	PR	CI (95%)	P
1st-degree relative with						
obesity	68 (21.5)	39 (16.9)	29 (34.1)	1.26	1.07-1.49	0.002
dyslipidemia	52 (16.5)	27 (11.7)	25 (29.4)	1.25	1.08-1.45	<0.001
diabetes	38 (12.0)	20 (8.6)	18 (21.2)	1.16	1.03-1.3	0.005
hypertension	74 (23.4)	45 (19.5)	29 (34.1)	1.22	1.04-1.44	0.010
heart disease/ischemic disease	32 (10.1)	18 (7.8)	14 (16.5)	1.1	0.99-1.22	0.040

PR: prevalence ratio; CI: confidence interval.

Table 4 presents the laboratory tests results, showing that 32.7% had low HDL, 18.4% had high total cholesterol, 11.4% had high LDL, and 32.0% had increased triglycerides. The excess weight group had significantly higher triglycerides (p = 0.017), glucose (p = 0.004), and C-reactive protein (p = 0.002).

**Table 4:** **Laboratory tests according to the BMI categories of individuals with congenital heart disease**

Variables	Underweight/Normal weight mean ± SD	Excess weight mean ± SD	p
Cholesterol	148.1 ± 25.6	154 ± 32.2	0.106
HDL	50.3 ± 11.3	48.9 ± 12.2	0.352
LDL	83.3 ± 21.7	89.3 ± 27.8	0.053
Triglycerides	72.6 ± 34.3	83.7 ± 38.8	0.017
Glucose	87 ± 9.5	90.6 ± 9.0	0.004
C-reactive protein	0.1 ± 0.1	0.2 ± 0.2	0.002
Hematocrit	39.1 ± 4.1	38.8 ± 3.4	0.523
Hemoglobin	13.3 ± 1.9	12.9 ± 1.5	0.099

SD: standard deviation.

## Discussion

The present study reports a high prevalence of excess weight in children and adolescents with congenital heart disease. Aditionally, we observed a high frequency of excess weight in parents and a positive family history for chronic non-transmissible diseases.

The prevalence of overweight and obesity in children with congenital heart disease was similar to that described in the literature for children with non-congenital disease [19, 21] In a population of patients with congenital heart disease in the U.S., researchers found a prevalence of more than 25% of obese and overweight children [22]. However, in a study published six years ago, the excess weight rate of a population of children and adolescents in Belgium was 7.6% [11].

In Brazil, the high prevalence of excess weight in children and adolescents in general has been a reason for concern, because other associated risk factors for ischemic heart disease, such as hypertension, glucose intolerance, dyslipidemia, and physical inactivity have emerged [7, 23–27].

The presence of modifiable risk factors for ischemic heart disease in this population, such as an abnormal lipid profile (high total cholesterol/LDL/triglycerides, low HDL) and excess weight may lead individuals with congenital heart disease to have a combination of risks that may persist into adulthood [4, 28]. These modifiable risk factors have been well discussed in the literature about children without heart disease [2, 21].

The presence of chronic diseases in families of patients with congenital heart disease is an additional risk factor for ischemic disease [7, 22, 28, 29], similarly to what occurs for healthy children/adolescents [23, 30] and adults in general [31, 32]. The presence of obesity in mothers in our study was directly related to their children’s excess weight. This findings could represent both biological/genetic characteristics and family lifestyles [14, 33, 34]. In a study comparing three generations of families, there was a strong significant relationship between the BMI of mothers and children, thus suggesting the discussion of inheritance of family patterns and lifestyle, as well as family phenotypes [14]. In another study evaluating the role of parents in the treatment of childhood obesity, the authors found that distorted maternal perception leads mothers to see their children’s excess weight as normal, making it difficult for them to admit their children need treatment [34].

In our study, approximately half of children and adolescents were irregularly active or sedentary. In many cases, physical activity may be limited by the parents anxiety [35].

Passive smoking was detected in almost half of the population studied, a rate much higher than in a survey conducted over the past decade, in which more than 25% of children lived with at least one smoking parent. Exposure to secondhand smoking in children causes higher rates of pneumonia, ear infections, sudden infant death syndrome, asthma, and other negative health effects [36]. In addition, children’s airways are more vulnerable, suffering dramatically with the effects of secondhand smoking [37]. Children exposed to tobacco smoke at a young age are more likely to become smokers and continue the cycle of smoking in adulthood [38].

It is important to consider that factors present since the children’s conception may contribute to “programming” of disease in adult life [39, 40]. The quality of the mother’s nutrition during pregnancy may affect the fetal metabolism and the child’s taste and attitudes towards food [41]. Along the life course, these factors interact with family habits and childhood risks to compose different health and disease pathways [14].

The present study has some limitations that deserve to be mentioned. Possible confounding biases may be related to memory bias and underreporting of information by the respondents. Cross-sectional designs do not allow causal inferences or detailed evaluation of sequences of events. Despite these limitations, to the best at our knowledge, this is one of the largest series of patients with congenital heart disease evaluated for these risk in Brazil or other developing countries.

## Conclusions

The obesity epidemic also affects children and adolescents with congenital heart disease. In this population, factors inherent to the heart disease can be added to other traditional risk factors for the development of ischemic heart disease in the future. Changes in the lifestyle are necessary to change these risk factors and its comorbidities in the adult life of these people who are living longer.

## Abbreviations

BMI: Body mass index
CHD: Congenital heart disease
DBP: Diastolic blood pressure
EDTA: Ethylenediaminetetraacetic acid
SBP: Systolic blood pressure.
